# Supplementary material for: Grain refinement in titanium prevents low temperature oxygen embrittlement
Source: Nat Commun. 2023 Feb 1;14:404. doi: 10.1038/s41467-023-36030-0 (PMC9892041; doi:10.1038/s41467-023-36030-0)
Supplement: Supplementary file 1 — Supplementary Information [file 41467_2023_36030_MOESM1_ESM.pdf]

Supplementary Information

Supplementary Table I Chemical compositions (wt.%) of pure Ti and Ti-0.3O alloy used in this study

wt%	Al	V	Fe	C	N	O
Pure Ti	0.002	0.003	0.002	0.003	0.001	0.018
Ti-0.3O	0.005	0.003	0.004	0.006	0.001	0.310

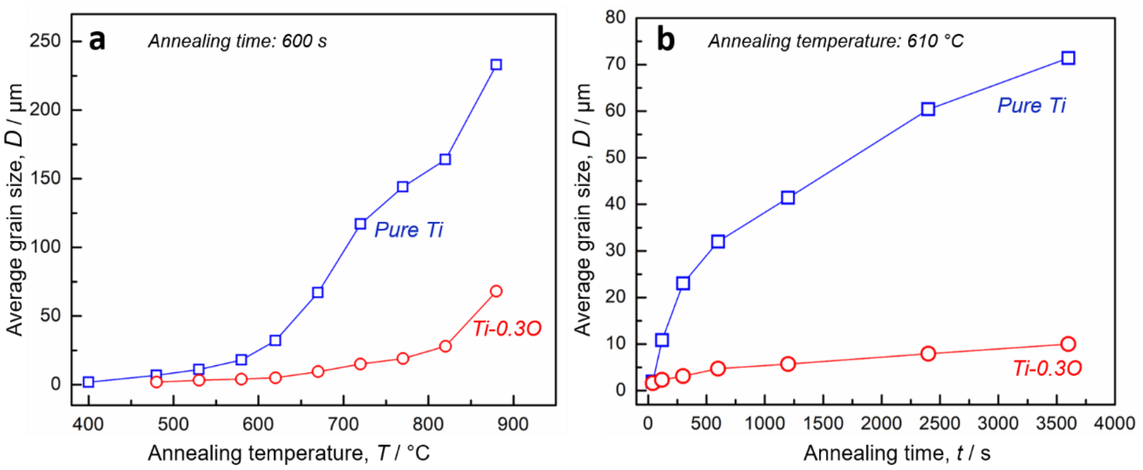

Supplementary Figure 1 The evolution of average grain size in pure Ti and Ti-0.3O alloys with annealing temperature **a** and time **b** after HPT deformed by 5 rotations. The average grain size of Ti-0.3O alloy were generally much smaller than those of pure Ti after the same annealing condition.

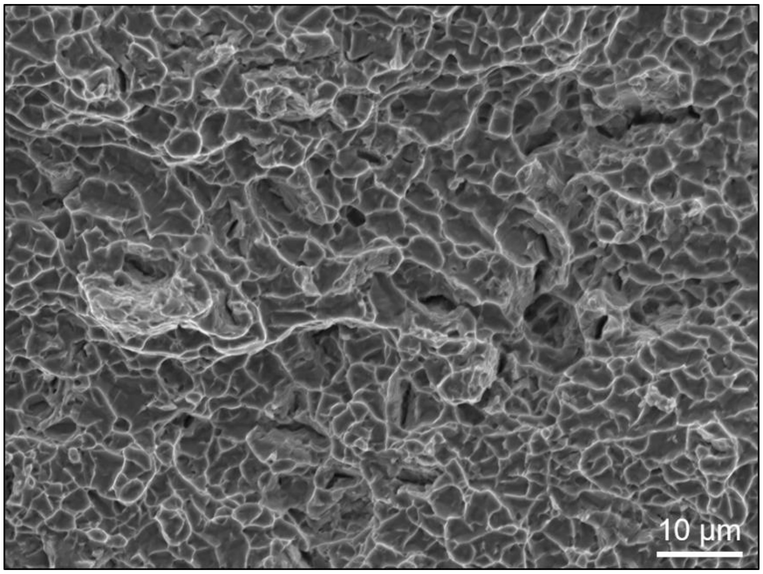

Supplementary Figure 2 Typical fracture tomography of UFG (D = 2.0 μm) Ti-0.3O alloy at 77 K. Dimple patterns were commonly observed in the fracture suraface, indicating a transgranular ductile fracture.

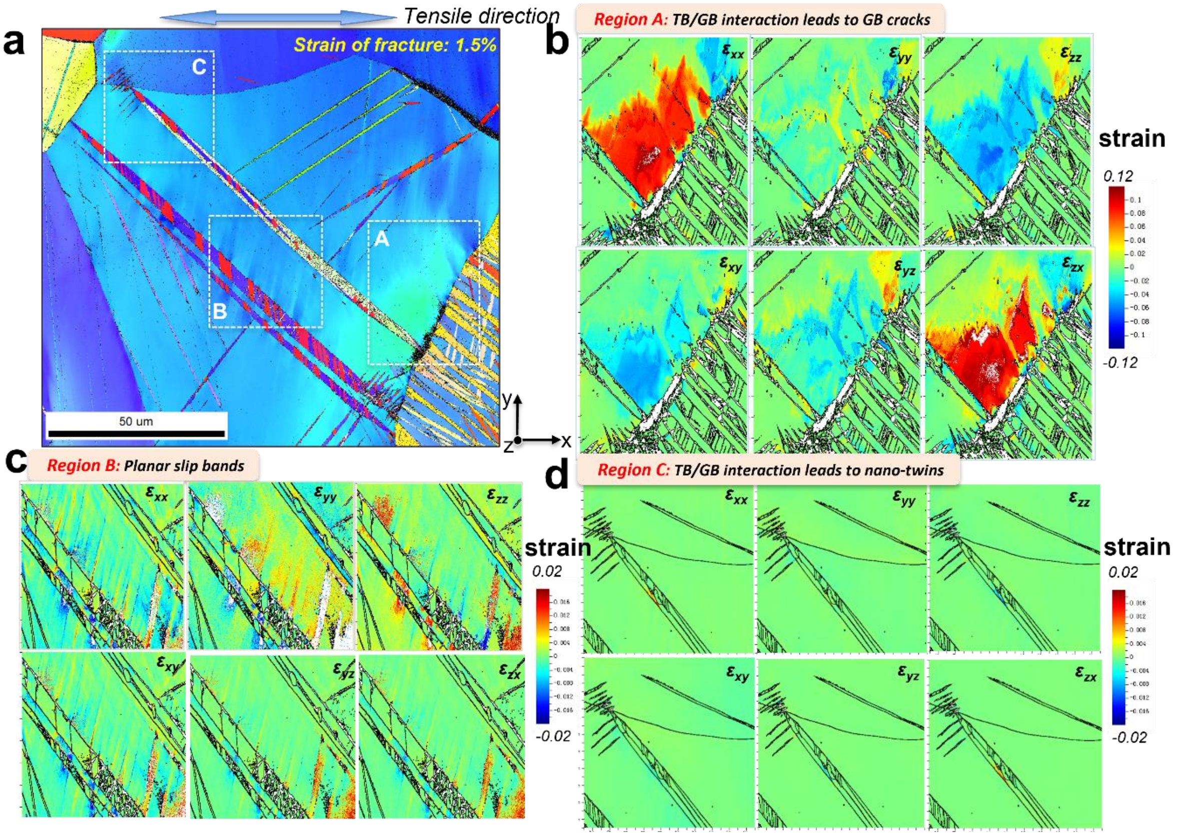

Supplementary Figure 3 All six strain tensors results of three representative regions in a coarse-grained (D = 68 μm) Ti-0.3O alloy **a** after tensile fractured at 77 K. **b** Region A: TB/GB interaction leads to formation of micro-cracks at grain boundary. **c** Region B: Planar slip bands. **d** Region C: TB/GB interaction leads to activation of nano-twins. The tensile direction is horizontal. The amount of elastic strain was indicated by the brightness of the color according to the scale bar.

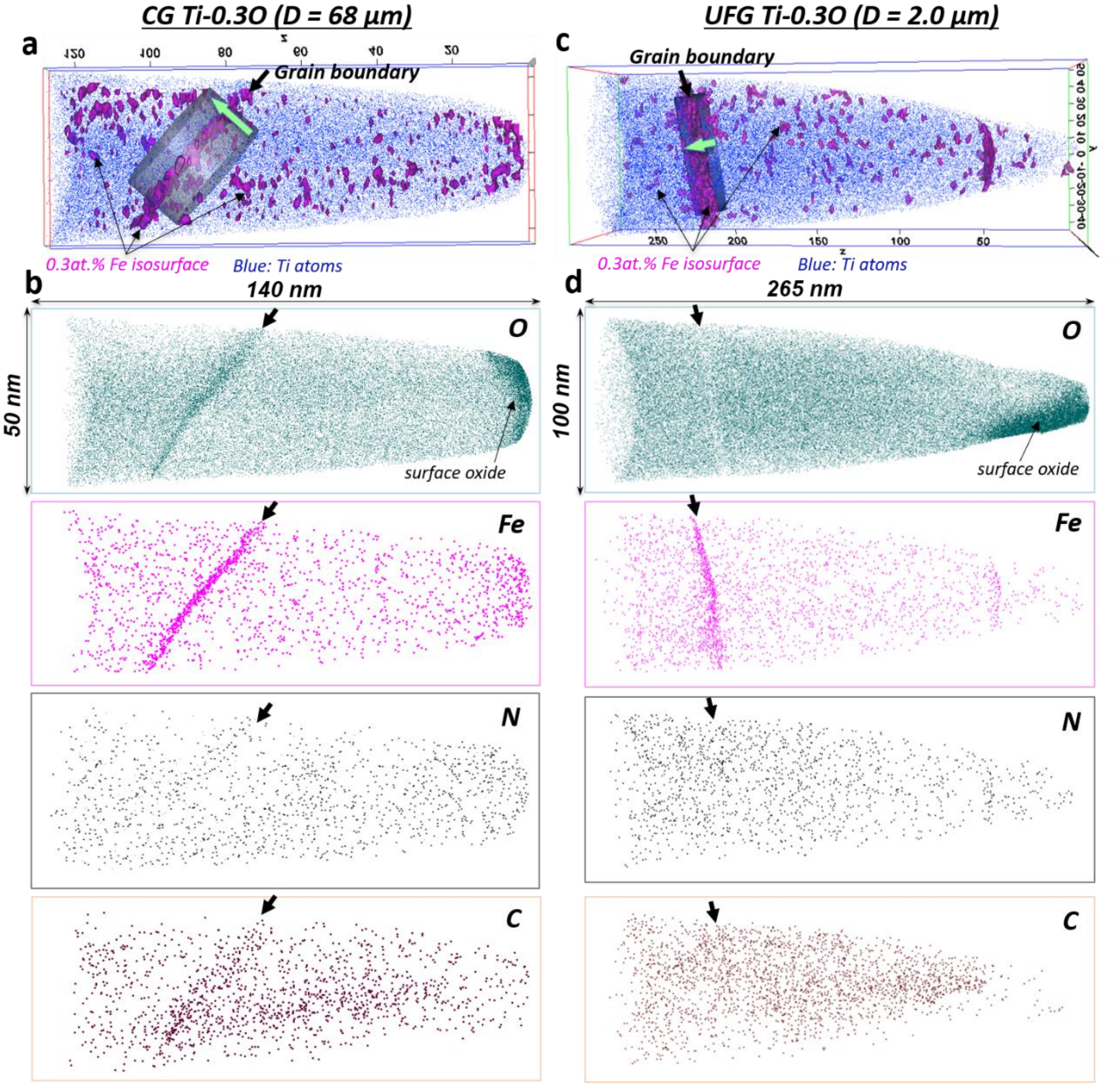

Supplementary Figure 4 APT results of CG (D = 68 μm) **a-b** and UFG (D = 2.0 μm) **c-d** Ti-0.3O alloy. The grain boundaries (**a** and **c**) in both samples were illustrated by the 0.3 at.% Fe isosurfaces (Ti atoms: blue). The atom maps of O, Fe, N and C in CG **b** and UFG **d** Ti-0.3O samples are shown, in which the locations of grain boundaries were indicated by arrows according to the TEM image as well as 0.3 at.% Fe isosurface.

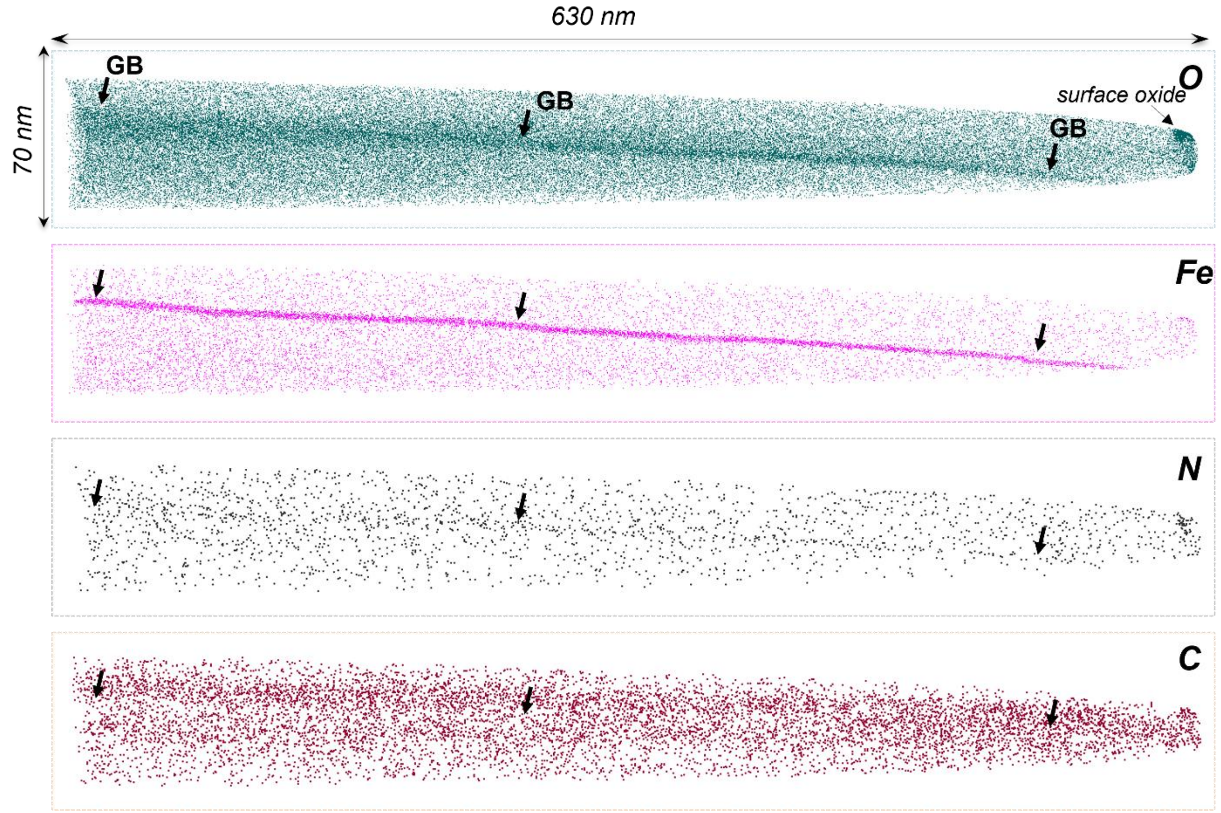

Supplementary Figure 5 Atom maps of trace elements in another APT specimen from CG (D = 68 μm) Ti-0.3O alloy, which contains a large portion of grain boundary that aligned nearly parallel to the tip. A clear grain boundary segregation of oxygen was also revealed in this specimen.

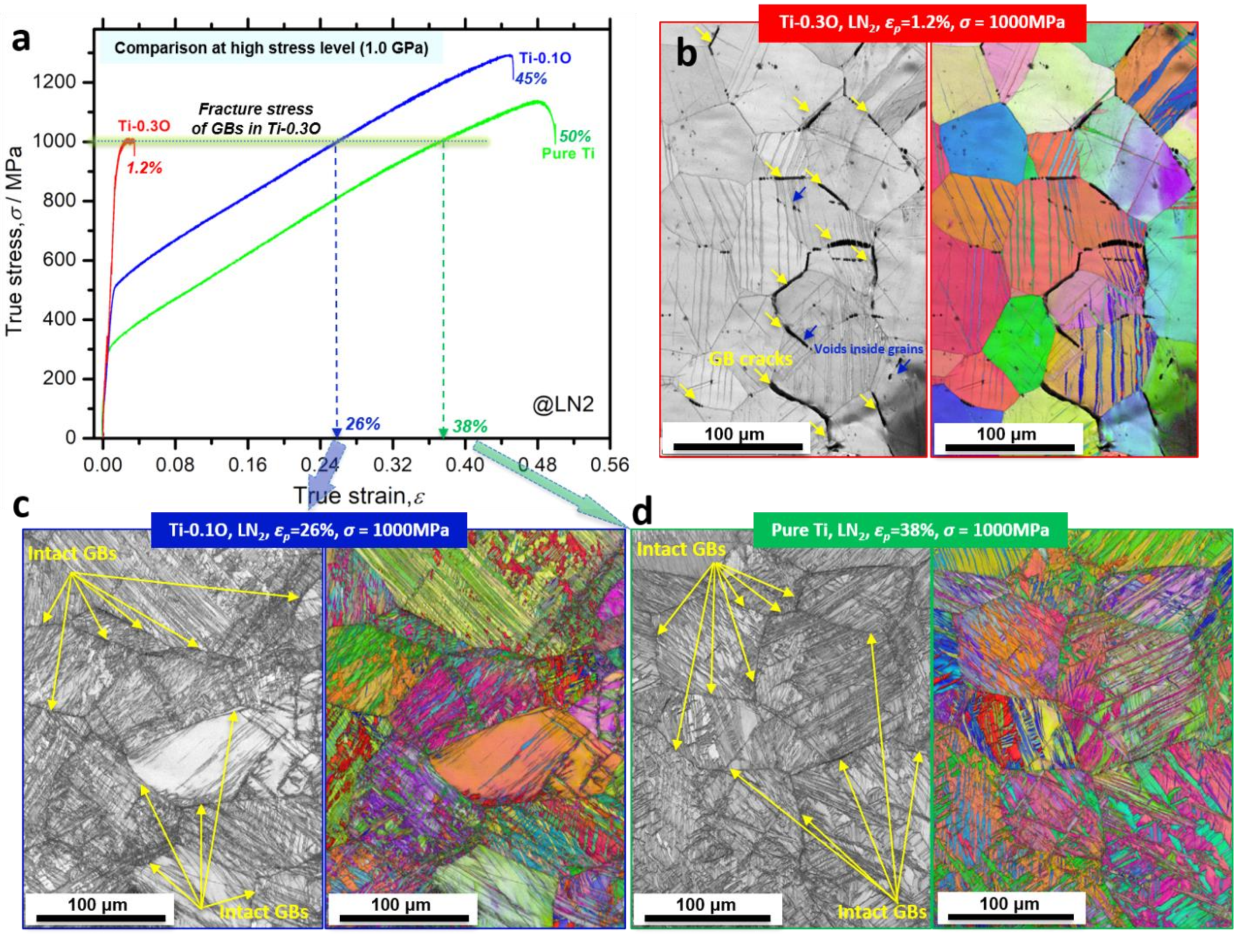

Supplementary Figure 6 a True stress-true strain curves of pure Ti, Ti-0.1O and Ti-0.3O alloys tensile deformed at 77 K. The tensile deformations were interrupted at the true stress of 1.0 GPa (roughly the fracture stress of Ti-0.3O) for all three microstructures for microstructure observation. The corresponding image quality (IQ) maps and IPF maps of Ti-0.3O (**b**), Ti-0.1O (**c**) and pure Ti (**d**) are shown, respectively. Compared with the easy fractured grain boundaries in Ti-0.3O (**b**), the grain boundaries in pure Ti (**d**) and Ti-0.1O (**c**) alloys were mostly intact at the same true stress level, indicating a higher grain boundary cohesion.

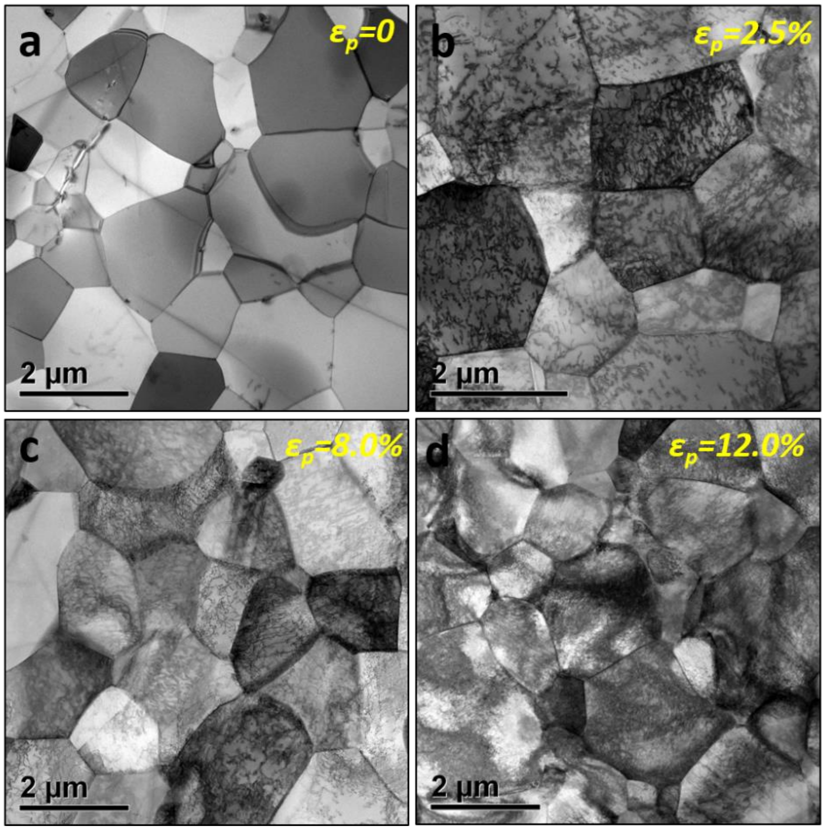

Supplementary Figure 7 STEM images showing dislocations in UFG Ti-0.3O (*D* = 2.0 µm) tensile deformed at 77 K by different plastic strains. **a** before deformation, **b** 2.5%, **c** 8.0% and **d** 12.0%.

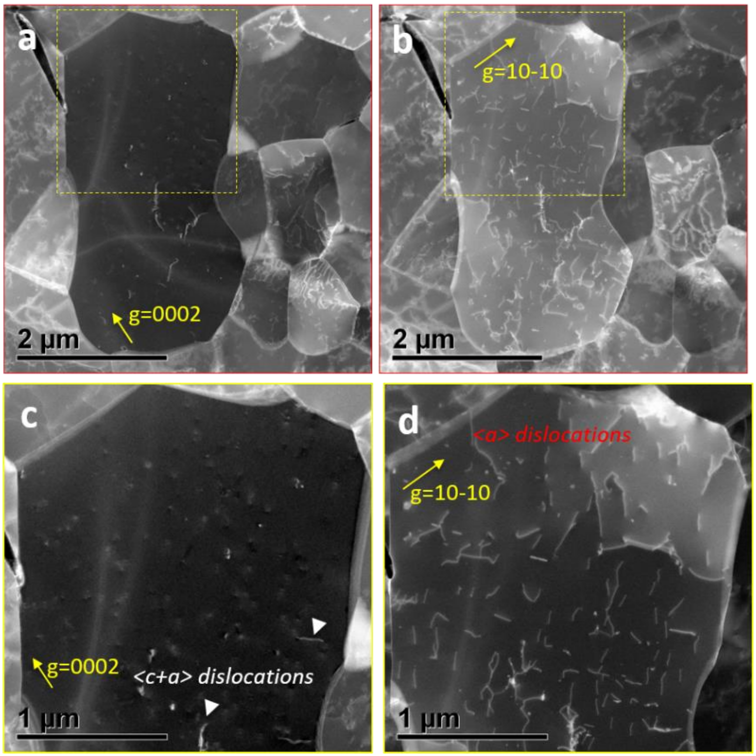

Supplementary Figure 8 Two-beam condition analysis of dislocations in UFG pure Ti deformed by 2.5% at 77 K. Most of the dislocations were confirmed to have only **<a>** component (**a** and **c**). The density of <**a**> dislocations were also relatively lower than that in the UFG Ti-0.3O counterpart (**b** and **d**).

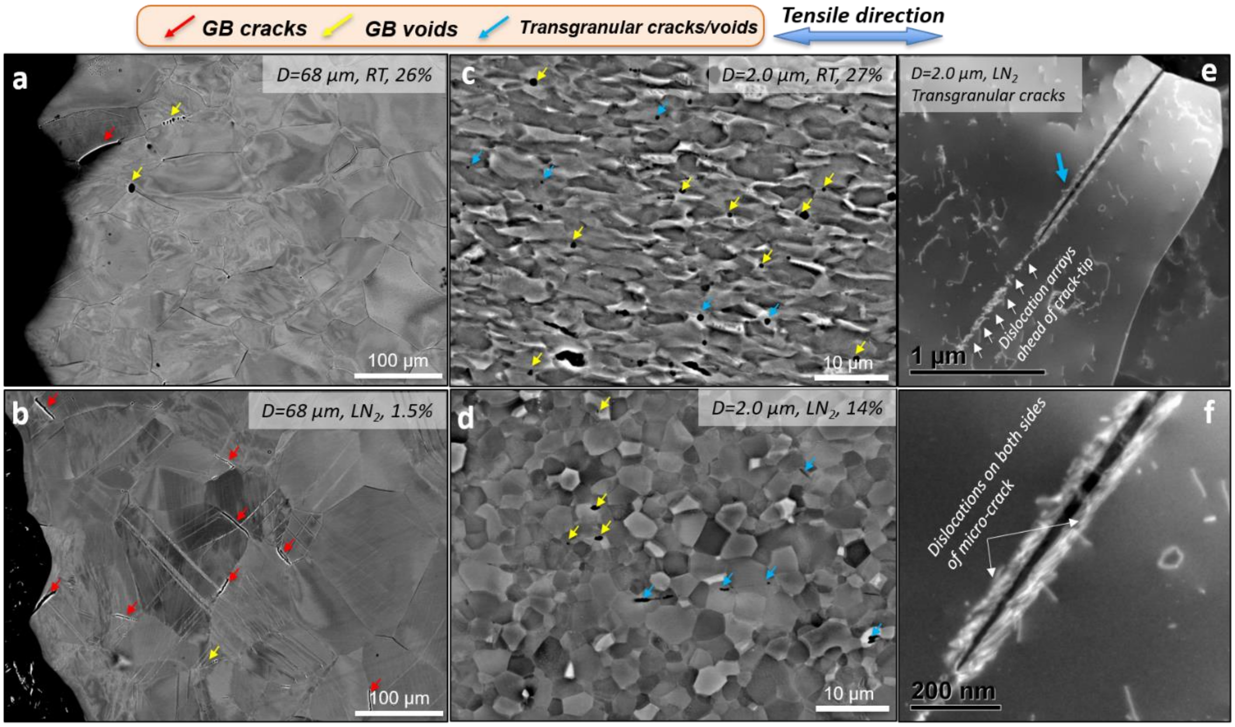

Supplementary Figure 9 Characterization of micro-cracks in CG (*D* = 68 µm) **a**-**b** and UFG (*D* = 2.0 µm) **c-f** Ti-0.3O alloy at room temperature (**a**, **c**) and liquid nitrogen temperature (**b**, **d**, **e**, **f**). In the CG sample, large GB-cracks became predominant at liquid nitrogen temperature (**b**). In the UFG sample, the poplutions of small GB-cracks and transgranular voids/cracks were much more close, regardless of the temperature (**c**, **d**). TEM image of transgranluar crack in UFG sample fractured at liquid nitrogen is shown in **e**. Dislocation arrays arrays were observed both ahead of the crack-tip (**e**) and on both sides of the micro-crack (**f**).

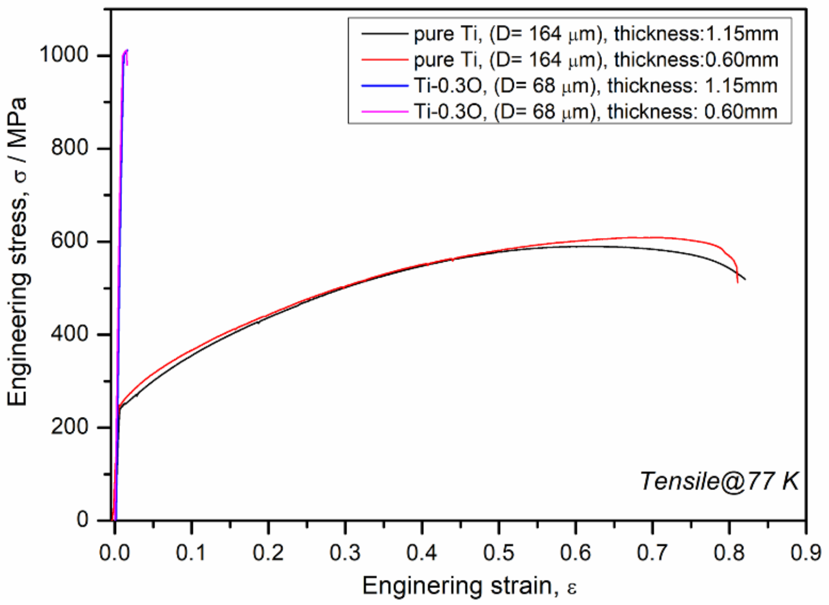

Supplementary Figure 10 Validation of the sample thickness effect on the tensile properties of coarse-grained pure Ti (D = 164 μm) and Ti-0.3O (D = 68 μm) alloys at 77 K. A good consistence was found for the tensile properties obtained from samples with different thickness of both alloys.

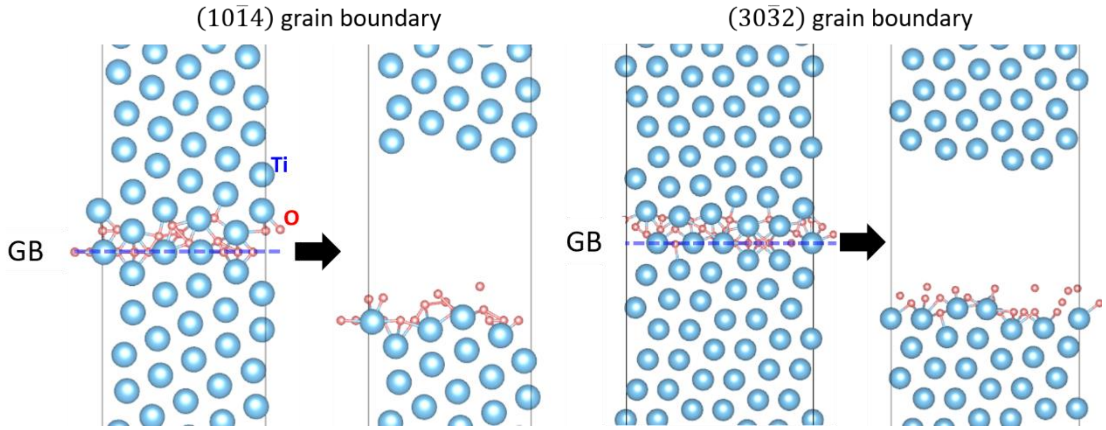

Supplementary Figure 11 DFT calculation set-ups of the grain boundary segregation and cleavage energies. All possible candidates of initial configuration for the interstitial oxygen sites at the grain boundary can be determined by Voronoi polyhedron analysis. DFT calculation was then carried out to explore the stable configuration for each interstitial site.
