# Supplementary material for: Grain refinement in titanium prevents low temperature oxygen embrittlement
Source: Nat Commun. 2023 Feb 1;14:404. doi: 10.1038/s41467-023-36030-0 (PMC9892041; doi:10.1038/s41467-023-36030-0)
Supplement: Supplementary file 3 — Description of Additional Supplementary Files [file 41467_2023_36030_MOESM3_ESM.pdf]

Description of Additional Supplementary Files

File Name: Supplementary Movie 1
Description: 3D reconstruction of dislocations in deformed coarse-grained Ti-0.3O specimen at 77 K viewed from X rotation.

File Name: Supplementary Movie 2
Description: 3D reconstruction of dislocations in deformed coarse-grained Ti-0.3O specimen at 77 K viewed from Y rotation.

File Name: Supplementary Movie 3
Description: 3D reconstruction of dislocations in deformed ultrafine-grained Ti-0.3O specimen at 77 K viewed from X rotation.

File Name: Supplementary Movie 4
Description: 3D reconstruction of dislocations in deformed ultrafine-grained Ti-0.3O specimen at 77 K viewed from Y rotation.
